# Knowledge, Attitude and Practice Regarding Cervical Cancer Screening Among Women Attending a Teaching Hospital, Bharatpur, Chitwan 

**Published:** 2017-03

**Authors:** Smita Shrestha, Prativa Dhakal

**Affiliations:** Chitwan Medical College, Bharatpur, Chitwan, Nepal

**Keywords:** Cervical Cancer, Screening, Knowledge and Practice

## Abstract

**Objective:** To find out the knowledge, attitude and practice regarding cervical cancer screening among women.

**Materials and methods:** A descriptive cross-sectional study design was used to collect data from 96 women. Each woman was selected alternately from Gynae Out-Patient Department of Teaching Hospital. Data was collected by using semi-structured interview schedule to find out knowledge and practice and Likert scale to find out the attitude regarding cervical cancer screening. Data was analyzed by using SPSS version 20.0 and interpreted in terms of descriptive and inferential statistics.

**Results:** Out of 96 women, mean age was 38.83 ± 6.57 and 90.6% respondents followed Hinduism. More than three fourth (85.4%) were literate and 59.4% were housewife. Only 9.4% were involved in cervical cancer prevention and screening awareness programme and 2.1% had family history of cervical cancer. As per the findings, only 34.4% and 27.8% had adequate knowledge and practice respectively whereas cent percent women had favorable attitude. Only education level of women was statistically significant with level of knowledge regarding cervical cancer screening (p = 0.041). There was strong negative correlation between knowledge score and practice score regarding cervical cancer screening among women (r = -0.194).

**Conclusion:** Considerable proportions of women have inadequate knowledge and practice regarding cervical cancer screening. Therefore cervical cancer screening health camps and awareness program should be conducted at community level for women to increase the level of knowledge and practice regarding cervical cancer screening.

## Introduction

Cervical cancer is an important public health problem in low income countries, where over 85% of the global deaths occur annually (1). According to a recent data, there were an estimated 527624 new cases and 265672 deaths from cervical cancer worldwide annually (2). 

Cervical cancer is the leading cancer and the leading cause of cancer deaths in women in developing countries. The highest incidence and mortality rates are in sub-Saharan Africa, Latin America, and South Asia. Overall, the mortality rates in developing countries are about four times (80-85%) than those in industrialized countries (3). Cervical cancer accounts for 7.5% of all female cancer deaths. Mortality varies 18-fold between the different regions of the world, with rates ranging from less than 2 per 100,000 in Western Asia, Western Europe and Australia/New Zealand to more than 20 per 100,000 in Melanesia (20.6), Middle (22.2) and Eastern (27.6) Africa (4). 

Cervical cancer is the most common cancer seen in Nepal. As per the findings of Hospital Based Cancer Registry from 2003-2012, 6249(20.9%) new cervical cancer cases has been diagnosed which is the highest among the total female new cancer cases (5). Though cervical cancer screening is available in some areas of Nepal, screening is mostly conducted when women come to the hospital for other medical problems and sometimes only when women present with symptoms. Despite the evidence that universal coverage is important, women in Nepal are not routinely screened before symptoms appear (6). The success and benefit of screening at a national level as a public health program to control and prevent cervical cancer depends to a great extent on the level of awareness of the potential beneficiaries (7). 

According to a study in Kathmandu 65.7% respondents had heard about cervical cancer. However, only 42.9% and 18.1% had knowledge about screening for cervical cancer and Pap smear test respectively. More than 85% of women had positive attitude towards screening but the practice of Pap smear test in the respondents was only 10.5% (8). 

The introduction of different screening techniques for cervical cancer has led to a significant reduction in morbidity and mortality from the disease. Various screening techniques have been introduced for early detection of cervical cancer in Nepal. For instance, Papanicolaou smear screening (cervical cytology) has been reported to be a good method for detecting early cervical cancer (1). According to a study, even though 90% of the respondents had heard about cervical cancer only 53% had heard about cervical cancer screening. Among 100 women 47% had adequate awareness, 38% had adequate attitude, and only 13% had adequate practice regarding cervical cancer screening (7). 

So, this study was conducted to find out the knowledge, attitude and practice regarding cervical cancer screening among women.

## Materials and methods

A descriptive cross sectional study was conducted on October 2016 in the Gynae outpatient department of Chitwan Medical College (CMC), Bharatpur, Nepal. The sample size was calculated by using the formula n = z^2^pq/d^2. ^From the calculation, the sample size was found to be 96. Every alternate sample was taken to meet the required sample size. Thus, the eligible women meeting the inclusion criteria i.e. age group of 30-60 years attending gynae OPD were interviewed. Ethical approval was obtained from CMC, Institutional Review Committee. Informed verbal consent was taken from women for participation. Confidentiality was maintained throughout the study. The interview schedule was comprised of four sections to gather information regarding sociodemographic characteristics, knowledge, attitude and practice regarding cervical cancer screening. Knowledge part consisted of 13 point scale. Each correct answer was given 1 score and 0 score for incorrect answer. The score was categorized asadequate knowledge (score of ≥ 50%) and inadequate knowledge (score of < 50%). Attitude was assessed by using a five point Likert scale ranging from strongly agree to strongly disagree containing 14 statements out of which 7 were positive statements and 7 were negative. The score was categorized as favorable attitude (score of ≥ 50%) and not favorable attitude (score of < 50%). Similarly practice score was categorized as adequate practice (score ≥ mean score i.e., ≥ 1.22) and inadequate practice (score < mean score i.e., < 1.22).

Pretesting was done among 10% of the total sample size and necessary modifications were made. Outcome variable was measured by assessing the level of knowledge, attitude and practice regarding cervical cancer screening. The independent variables were age, education, religion, occupation, family history of cervical cancer, peer influence, influence of media, involvement in awareness program on cervical cancer screening, previous exposure to cervical cancer screening and availability of health service. SPSS 20 version was used for statistical analysis. Descriptive statistics was used to calculate frequency, percentage, mean and standard deviation. Chi-square test was used to find out the association between the independent and dependent variables. The level of significance is 0.05.

## Results

Table 1 shows out of 96 respondents, more than half (58.3%) were in age between 30-39 years with mean38.83. Majority (90.6%) of the respondents followed Hindu religion.

**Table 1:** Respondents’ Socio-demographic Characteristics (n = 96)

**Variables **	**Frequency (%)**
Age (years)	
30-39	56 (58.3)
40-49	30 (31.3)
50 and above	10 (10.4)
Religion	
Hinduism	87 (90.60)
Buddhism	6 (6.3)
Christianity	2 (2.1)
Islam	1 (1.0)
Education status	
Literate	82 (85.4)
Illiterate	14 (14.6)
If literate, education level (n=82)	
Read and write	4 (4.9)
Basic education level	21 (25.6)
Secondary education level	29 (35.4)
Higher secondary and above	28 (34.1)
Occupation	
House maker	57 (59.4)
Agriculture	10 (10.4)
Business	15 (15.6)
Job holder	14 (14.6)
Age of marriage (years)	
<15	3 (3.1)
15-19	37 (38.5)
20-24	46 (47.9)
25-29	9 (9.4)
>30	1 (1.0)

More than three fourth (85.4%) of the respondents were literate, among them, 35.4% had completed secondary level of education. Regarding occupation, more than half (59.4%) of the respondents were house maker. Almost half (47.9%) of the respondents were married at age between 20-24 years with mean ± SD (Min, Max) age of marriage was 20.08 ± 3.420 (12, 33).

Table 2 shows that more than half (56.3%) of the respondents had knowledge regarding meaning of cervical cancer screening. More than two third (68.8%) of the respondents were aware that Pap smear is a screening test for cervical cancer. Almost one fourth (24.0%) of the respondents agreed to do the test if they were given opportunity.

Table 3 shows more than three fourth (77.8%) had performed cervical cancer screening for diagnostic purpose. Majority (92.1%) of the respondents said I’m not ill, so it is not necessary as the reason for not performing cervical cancer screening.

Table 4 shows only 34.4% had adequate knowledge regarding cervical cancer screening with mean ± SD (Min, Max) 1.04 ± 3.863 (1, 22). 

Out of 96 respondents, cent percent had favorable attitude regarding cervical cancer screening. Out of 96 respondents, only 18 (18.8%) had performed cervical cancer screening at least once.

**Table 2:** Knowledge and Attitude regarding Cervical Cancer Screening

**Variables **	**Frequency (%)**
Knowledge about cervical cancer screening	
Meaning of cervical cancer screening	54 (56.3)
Type of cervical cancer screening	64 (68.8)
Age for screening	24 (25.0)
Best time for cervical cancer screening	32 (33.3)
Time interval for screening	7 (7.3)
Attitude regarding cervical cancer	
Cervical cancer screening is good for early detection of cervical cancer	22 (22.9)
Cervical cancer screening test is done for all gynecologic cancers	
All women above 30 years need to be screened for cervical cancer	7 (7.3)
Women with only one sexual partner should also have cervical cancer screening	20 (20.8)
Women with total hysterectomy can stop getting screened	17 (17.7)
Women who have stopped having children can stop cervical cancer screening	
Women who have received HPV vaccine don’t need cervical cancer screening	11 (11.5)
Women above 65 years do not need cervical cancer screening	4 (4.2)
Cervical cancer screening is painful	
It is time consuming test	5 (5.2)
Cervical cancer screening destroys the ability of a woman to have a baby	
Cervical cancer screening enlarges the vagina and reduces sexual enjoyment	9 (9.4)
I have thought of doing cervical cancer screening	10 (10.4)
If I am explained about cervical cancer screening and opportunity is given to do the test, I will be willing to do that test	10 (10.4)

**Table 3:** Respondents’ Reasons for Performing and Not Performing Cervical Cancer Screening

**Variables **	**Frequency**	**Percentage**
Reason for performing screening* (n = 18)		
Preventive measure	1	5.6
Diagnostic purpose	14	77.8
Health worker recommendation	3	16.7
Arranged health camps	4	22.2
Reason for not performing screening* (n = 78)		
I’m not ill, so it is not necessary	70	92.1
Fear of the procedure	2	2.6
Not recommended	18	23.7
No knowledge	6	7.9
Embarrassment	4	5.3
Husband disapproves of cervical cancer screening	3	3.9
No access to clinic for screening	1	1.3

* Multiple response

**Table 4:** Respondents’ Level of Knowledge and attitude regarding Cervical Cancer Screening

**Level **	**Adequate n (%)**	**Inadequate n (%)**
Knowledge	33 (34.4)	63 (65.6)
Practice	5 (27.8)	13 (72.2)

Among them, only 27.8% had adequate practice regarding cervical cancer screening with mean ± SD (Min, Max) 1.22 ± 1.309 (1, 4).

Table 5 illustrates that only education level of respondent is significantly associated with the level of knowledge regarding cervical cancer screening (p < 0.05).

Table 6 illustrates that there was strong negative correlation between knowledge score and practice score regarding cervical cancer screening among women. 

**Table 5:** Association between Level of Knowledge regarding Cervical Cancer Screening and Selected Socio-demographic Variable (n = 96)

**Variables**	**Level of knowledge**		**p value**
	**Adequate n (%)**	**Inadequate n (%)**	
Age			
30-39 years	20(35.7)	36(64.3)	0.930
40-49 years	10(33.3)	20(66.7)	
50 years and above	3(30.0)	7(70.0)	
Education level			
Illiterate	1 (7.1)	13 (92.9)	0.014
Basic education	7 (28.0)	18 (72.0)	
Secondary level and above	25 (43.9)	32 (56.1)	
Occupation			
Unemployed	22 (32.8)	45 (67.2)	0.629
Employed	11 (37.9)	18 (62.1)	
Age of marriage			
Below 20 years	12 (30.0)	28 (70.0)	0.446
20 years and above	21 (37.5)	35 (62.5)	
Husband education level (n=87)			
Basic education	6 (42.9)	8 (57.1)	0.678
Secondary level and above	27 (37.0)	46 (63.0)	
Husband occupation (n=94)			
Agriculture	3 (16.7)	15 (83.3)	0.08
Business	6 (25.0)	18 (75.0)	
Job holder	13 (46.4)	15 (53.6)	
Others	11 (45.8)	13 (54.2)	
Involvement in awareness program			
Yes	4 (44.4)	5 (55.6)	0.765
No	29 (33.3)	58 (66.7)	

**Table 6:** Correlation between Knowledge and Practice Score regarding Cervical Cancer Screening

**Variables**	**R**	**p value**
Knowledge score Vs. Practice score	-0.194	0.440

r = Karl Pearson’s Correlation Coefficient.

## Discussion

As per the findings, 56.3% knew the reason for screening and 68.8% knew Pap smear test as a screening test. The finding is inconsistent with the study conducted by Shrestha (2014) that showed 53.0% knew the reason for screening and Pap smear as a screening test (7). Accordingly, 68.8% and 21.5% mentioned Pap smear and Visual Inspection with Acetic Acid (VIA) as the screening test for cervical cancer. The finding is inconsistent with the finding of John (2011) which showed 2.9% and 11.3% mentioned Pap smear and VIAas the screening test for cervical cancer respectively (9). Similarly, 25.0% knew the correct age to start screening and 7.3% knew that screening should be done in every 3 years. The finding is inconsistent with the study of Shrestha (2014) that showed 16.0% knew the correct age for screening and 21.0% knew that screening should be done in every 3 years (7). Accordingly, 33.3% knew screening should be done 10-20 days of menstruation which was similar with the finding of Shrestha (2014) that showed 34.0% knew that screening should be done 10-20 days of menstruation (7).

When asked about willingness to perform cervical cancer screening, 68.8% and 24.0% strongly agreed and agreed to undergo cervical cancer screening respectively. The finding is inconsistent with the finding of Mbamara et al. (2011) that showed 57.1% of the women agreed that they would like to undergo a cervical cancer screening test (10).

According to the findings, 33.3%, 22.2% and 16.7% had performed cervical cancer screening once, twice and three times respectively. The finding is inconsistent with the findings of Chamani et al. (2012) which showed 88.4%, 9.5% and 1.0% performed cervical cancer screening once twice and three times respectively (11).

The most common reason for participating in cervical cancer screening was for diagnostic purpose and the most common reason for non-participation was participant was not ill, so it was not necessary. The finding is inconsistent with the study conducted by Chamani et al. (2012) that showed physicians’ or other health care workers’ advice as reason for participating and no physicians’ recommendation and lack of knowledge as reason for not participating (11).

This study revealed that only 34.4% had adequate knowledge regarding cervical cancer screening. The finding is similar with the finding of Singh et al. (2014) that showed 32.7% of the respondents had adequate knowledge. As per the finding of the study, cent percent had favorable attitude. This finding is inconsistent with the study conducted by Singh et al. (2014) in which 18.2 % had favorable attitude. With respect to practice, 72.2% had inadequate practice. The finding is similar with the study conducted by Singh et al. (2014) that is 80.4%. (12). Statistical significant association was found between education level and level of knowledge regarding cervical cancer screening (p = 0.014). This study is supported by Obi (2015) that showed the association between level of education and level of knowledge regarding cervical cancer screening (p = 0.013) (12).

## Conclusion

Two third of the women have inadequate knowledge. Cent percent women have favorable attitude towards cervical cancer screening and almost three fourth of the women need to improve practice regarding cervical cancer screening. The level of knowledge regarding cervical cancer screening is influenced by the education level of women. There is strong negative correlation between knowledge score and practice score regarding cervical cancer screening among women. Therefore, there is need to conduct awareness program on cervical cancer screening to increase the level of knowledge and practice regarding cervical cancer screening through the medium of health personnel, friends and mass medias like television, radio and newspaper as these are the common source of information. Cervical cancer screening health camps can also be conducted at the community level.
